# Targeted alpha therapy using a novel CD70 targeted thorium-227 conjugate in *in vitro* and *in vivo* models of renal cell carcinoma

**DOI:** 10.18632/oncotarget.16910

**Published:** 2017-04-07

**Authors:** Urs B. Hagemann, Dessislava Mihaylova, Steinar R. Uran, Joergen Borrebaek, Derek Grant, Roger M. Bjerke, Jenny Karlsson, Alan S. Cuthbertson

**Affiliations:** ^1^ Thorium Conjugate Research, Bayer AS, Oslo, Norway

**Keywords:** targeted alpha therapy (TAT), alpha particles, radioimmunotherapy, thorium-227, renal cell carcinoma

## Abstract

The cell surface receptor CD70 has been previously reported as a promising target for B-cell lymphomas and several solid cancers including renal cell carcinoma. We describe herein the characterization and efficacy of a novel CD70 targeted thorium-227 conjugate (CD70-TTC) comprising the combination of the three components, a CD70 targeting antibody, a chelator moiety and the short-range, high-energy alpha-emitting radionuclide thorium-227 (^227^Th). *In vitro* analysis demonstrated that the CD70-TTC retained binding affinity to its target and displayed potent and specific cytotoxicity compared to an isotype control-TTC. A biodistribution study in subcutaneous tumor-bearing nude mice using the human renal cell carcinoma cell line 786-O demonstrated significant uptake and retention with 122 ± 42% of the injected dose of ^227^Th per gram (% ID/g) remaining in the tumor seven days post dose administration compared to only 3% ID/g for the isotype control-TTC. Tumor accumulation correlated with a dose dependent and statistically significant inhibition in tumor growth compared to vehicle and isotype control-TTC groups at radioactivity doses as low as 50 kBq/kg. The CD70-TTC was well tolerated as evidenced by only modest changes in hematology and normal gain in body weight of the mice. To our knowledge, this is the first report describing molecular targeting of CD70 expressing tumors using a targeted alpha-therapy (TAT).

## INTRODUCTION

The principle of targeted alpha therapy in oncology is based on the specific delivery of high linear energy transfer (LET) alpha-particle emitting radionuclides to tumor-associated antigens [1, 2]. Several alpha radionuclides including bismuth-213 (^213^Bi; t_1/2_ = 46 min), astatine-211 (^211^At; t_1/2_ = 7.2 hours), actinium-225 (^225^Ac; t_1/2_ = 9.9 days) and thorium-227 (^227^Th; t_1/2_ = 18.7 days) combined with monoclonal antibodies have been previously reported in the literature to be highly efficacious, [3–8] lending further support for the continued clinical development of this new modality in cancer therapy. The short range of approximately 50 to 80 μm in tissue of the alpha-particle has the potential to reduce off-target toxicity compared to either external beam radiation therapy (EBRT) or the longer range beta-emitters such as yttrium-90 (^90^Y) or lutetium-177 (^177^Lu).

The recent approval of the first-in-class alpha radiopharmaceutical Xofigo (radium-223 dichloride, ^223^RaCl_2_) for the treatment of metastatic castration resistant prostate cancer [3] by the Food and Drug Administration (FDA) has further increased the interest in new applications of TAT. Although the inherent bone-seeking accumulation of radium-223 (^223^Ra) [4] allows for effective delivery to bone metastases, the lack of suitable chelating agents has so far impeded the development of ^223^Ra-based radioimmunoconjugates for broader applications in oncology [5]. In contrast, ^227^Th, the parent radionuclide of ^223^Ra, can be readily complexed to chelators such as the octadentate 3,2 hydroxypyridinone (3,2-HOPO) class [6]. These 3,2-HOPO chelators can be chemically conjugated to tumor-targeting monoclonal antibodies (mAbs) enabling radiolabeling at ambient temperature. Thorium-227 has a half-life of 18.7 days comparable to the blood half-life of therapeutic antibodies in humans and decays releasing a total of five high-energy alpha-particles, through the progeny cascade ^223^Ra, ^219^Rn, ^215^Po, ^211^Pb,^211^Bi and ^207^Tl before reaching stable ^207^Pb [13]. The targeted thorium-227 conjugates (TTCs) have the potential to deliver highly potent alpha-particles to many different cancer types including both solid tumors and those of the lympho-hematopoietic system [7, 8].

The cell surface receptor CD70 and its ligand CD27L are members of the TNF ligand and receptor family [9]. In healthy individuals the ligand-receptor pair plays an important role in T-cell signalling and CD70 expression occurs transiently on activated T-cells, Toll-like receptor (TLR)-stimulated B-cells, mature dendritic cells (DCs), natural killer (NK) cells and on dendritic and epithelial cells of the thymic medulla [10–15]. The expression of CD70 and CD27L is highly regulated and limited to hematopoetic cells. CD70 is overexpressed on both T and B-cell lymphomas with the highest incidence in diffuse large B-cell lymphoma (DLBCL) [16]. In solid tumors CD70 expression was first described in nasopharyngeal carcinoma potentially associated with Epstein-Barr virus (EBV) infection [17, 18]. Subsequently, CD70 has been found to be overexpressed in renal cell carcinoma (RCC) [19–21], adenocarcinoma of pancreas and ovaries, breast and colon cancer, glioblastoma, laryngeal carcinoma and melanoma [15, 22, 23].

Due to its restricted and highly regulated expression on hematopoietic cells and healthy tissues as well as its internalizing properties, molecular targeting of CD70 positive tumors has been reported in several preclinical and clinical studies using antibodies inducing antibody-dependent cell-mediated cytotoxicity (ADCC) and complement-dependent cytotoxicity (CDC) as well as antibody drug conjugates (ADCs). ARGX-110, a glyco-engineered fully humanized monoclonal antibody was shown to induce potent ADCC upon binding to CD70 positive tumor cells [24]. The safety and tolerability profile of this antibody is currently being tested in a Phase 1 trial in patients with advanced malignancies (see clinical trial number NCT01813539). Further, the antibody SGN-70 has both inherent ADCC and CDC activity, demonstrating *in vitro* and *in vivo* efficacy in preclinical models of Non-Hodgkin Lymphoma (NHL) and multiple myeloma [21, 25]. The ADC, SGN-75, conjugated with the microtubule inhibitor auristatin, has also demonstrated potent activity in preclinical tumor models [26–29] and was the subject of a clinical trial in CD70-positive NHL and metastatic RCC patients (see clinical trial number NCT01015911). Further optimization led to SGN-CD70A in which the cytotoxic payload was the DNA cross-linking pyrrolobenzodiazepine (PBD) dimer [30]. This conjugate also demonstrated potent *in vitro* and *in vivo* activity, in models of NHL and RCC. SGN-CD70A is currently being tested in a Phase 1 study (see clinical trial number NCT02216890). Finally, the CD70-targeted ADC, MDX-1203/BMS-936561, conjugated to a DNA cross-linking alkylating agent has also been tested in the clinic (see clinical trial number NCT00944905) showing both evidence of efficacy and an acceptable safety profile at doses up to 8 mg/kg in patients suffering from NHL and clear cell RCC [31].

We present herein for the first time a CD70 monoclonal antibody bearing the thorium-227 payload. The resulting CD70-targeted thorium-227 conjugate (CD70-TTC) was found to possess both potent *in vitro* activity and demonstrated significant inhibition of tumor growth *in vivo* in the human renal cancer 786-O cell line derived xenograft model providing further support for the development novel radiotherapeutics for this important target.

## RESULTS

### Generation and characterization of the CD70-TTC

**Figure 1:**
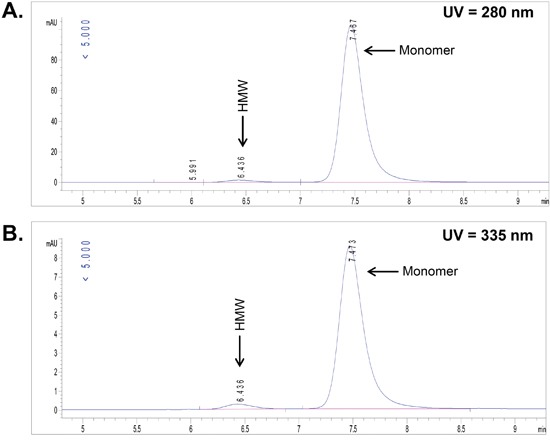
Determination of chelator to antibody ratio (CAR) by UV-size-exclusion chromatography UV-absorbance for the CD70 antibody-chelator conjugate at 280 nm **(A)** and the UV-absorbance of the chelator within the CD70 antibody-chelator conjugate at 335 nm **(B)** were monitored in parallel.

The CD70 targeting monoclonal antibody (IgG1) was conjugated with the 3,2-HOPO chelator through the ε-amino groups of lysine residues using water soluble carbodiimide as previously described [6, 8]. The chelator to antibody ratio (CAR) was determined using size-exclusion chromatography (SEC) by simultaneously monitoring the UV absorbance of the protein at 280 and 335 nm. The resulting conjugate was determined to have a CAR of 1.0, a high-molecular weight (HMW) fraction of 2 % and a monomeric peak of 98 % as shown in Figure 1.

**Figure 2:**
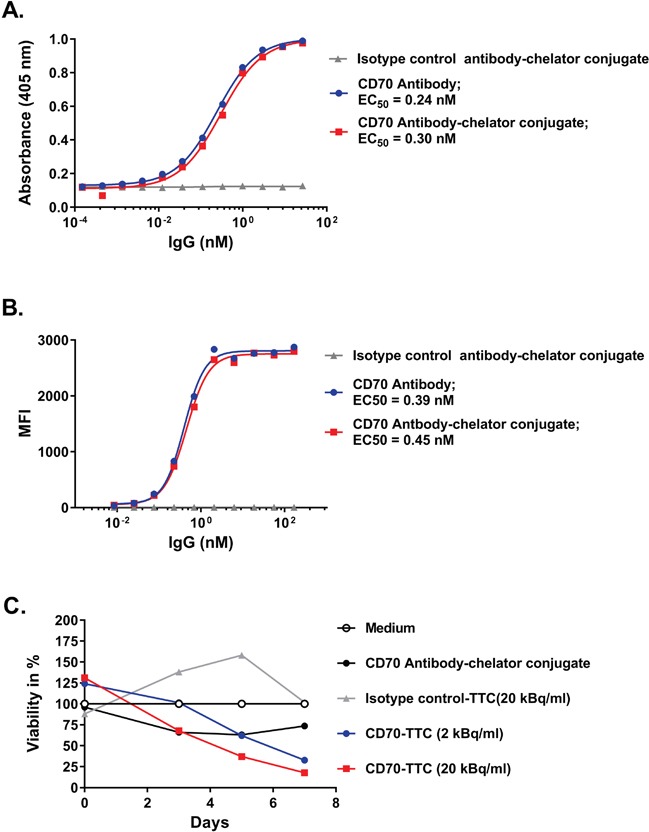
Comparison of binding potencies in ELISA, flow cytometry and *in vitro* cytotoxicity **(A)** ELISA on immobilized recombinant human CD70, comparing CD70 antibody, CD70 antibody-chelator conjugate and isotype control antibody-chelator conjugate. EC_50_ values are given in nM. **(B)** Flow cytometry analysis on human 786-O cells, comparing CD70 antibody with CD70 antibody-chelator conjugate and an isotype control antibody-chelator conjugate. EC_50_ values are given in nM. **(C)** *In vitro* cytotoxicity performed using CellTiter-Glo® assay for CD70-TTC and isotype control-TTC, radiolabeled at a specific activity of 50 kBq/μg, on 786-O cells at radioactive concentrations of 2 and 20 kBq/ml. At study days 3, 5 and 7, cell viability was measured and normalized to cells incubated in medium only. A non-radiolabeled CD70 antibody-chelator conjugate was included for comparison.

The binding properties of the CD70 antibody-chelator conjugate were compared to the naked CD70 antibody by ELISA using recombinant human CD70. EC_50_ values of 0.24 nM for the CD70 antibody and 0.3 nM for the CD70 antibody-chelator conjugate were determined (Figure 2A) indicating that the binding potency was not impaired by conjugation. Similarly, binding to the CD70 expressing human renal cancer cell line 786-O was determined by FACS analysis as shown in Figure 2B with EC_50_ values determined to be 0.39 nM and 0.45 nM for the CD70 antibody and CD70 antibody-chelator conjugate respectively.

Radiolabeling of the CD70 antibody-chelator conjugate was effected by simple addition of a solution of the conjugate to a vial containing a thorium-227 film at ambient temperature as described previously [8]. The resulting radiolabeled product was analyzed by instant thin-layer chromatography (iTLC) and radiochemical purity (RCP), defined as the fraction of bound ^227^Th in the CD70-TTC compared to free ^227^Th, was determined to be consistently ≥ 95 %.

### *In vitro* cytotoxicity of the CD70-TTC

*In vitro* cytotoxicity was performed using the CellTiter-Glo® assay on CD70-positive 786-O cells. Cells were exposed to the CD70-TTC at concentrations of 2 and 20 kBq/ml and to the isotype control-TTC at 20 kBq/ml. Both conjugates were radiolabeled at a specific activity of 50 kBq/μg. As presented in Figure 2C, exposure of cells to CD70-TTC demonstrated a dose-dependent reduction in cell viability over time. The effect was also highly specific to the CD70-TTC. These data demonstrate that CD70-TTC induces potent and specific reduction in cell viability.

### Biodistribution of the CD70-TTC

**Figure 3:**
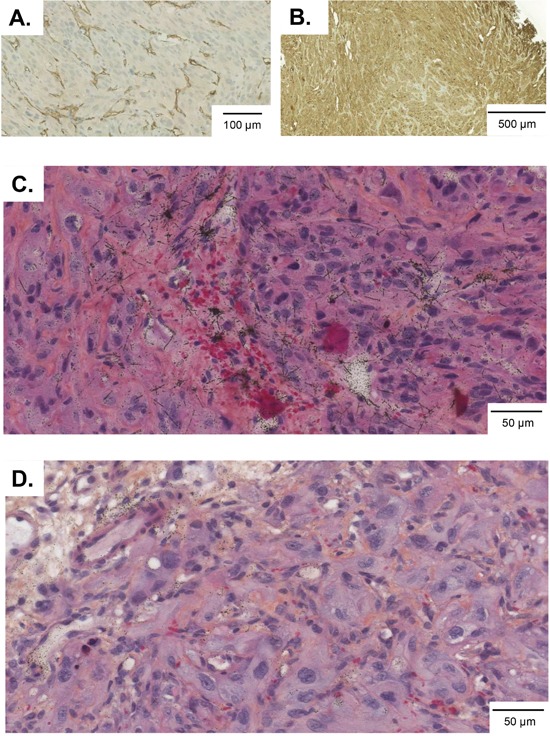
786-O tumor sections from animals in the biodistribution study Frozen tumor sections were prepared seven days after dose administration and analyzed for microvessel density **(A)**, CD70-expression **(B)** and autoradiography **(C)** and **(D).** Alpha-tracks in “star-like formations” were observed in tumor sections treated with CD70-TTC (C), but not in tumors treated with isotype control-TTC (D).

Biodistribution of the CD70-TTC was performed in 786-O tumor-bearing athymic mice bearing lesions with an average dimension of 200-300 mm^3^. The mice received an intravenous injection of CD70-TTC at a radioactive dose of 500 kBq/kg at a fixed protein dose of 0.36 mg/kg. To demonstrate specificity, a second group of animals were treated at the same radioactive and protein dose levels (500 kBq/kg; 0.36 mg/kg) with the non-targeting isotype control-TTC. Immunohistochemical analysis of the tumors demonstrated high (3+) CD70 target expression and an average micro vessel density (MVD) of 324 ± 55 per mm^2^. Percentage of necrotic areas was in the range of not detectable to 14 % of the analyzed tumor sections (Figure 3A and 3B). Autoradiography was performed on tumor sections after dose administration, the alpha-particle tracks were visualized as star-like formations as shown in Figure 3C and were not observed in tumor sections derived from the isotype control (Figure 3D) further supporting the specificity of the CD70-TTC.

**Figure 4:**
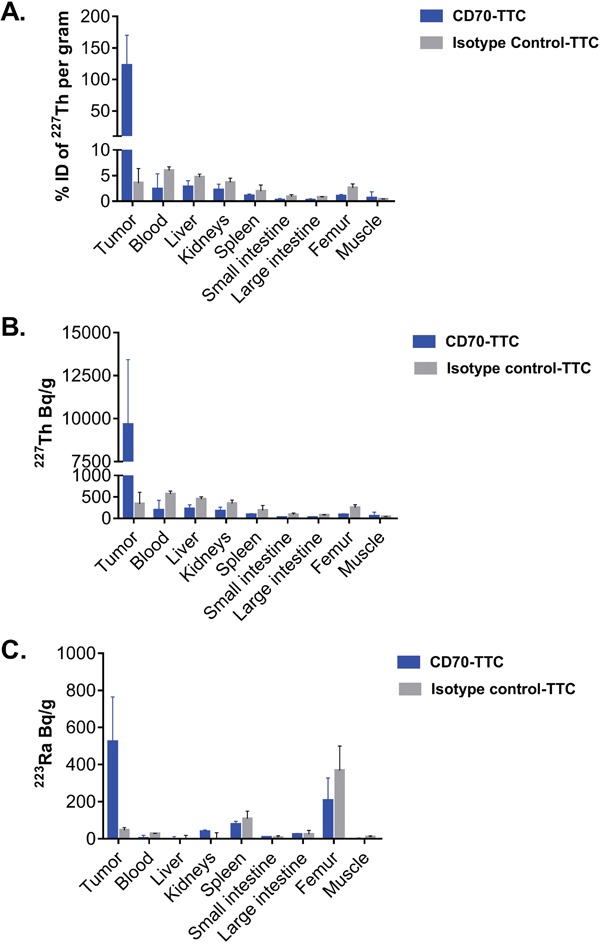
Biodistribution of CD70-TTC in the subcutaneous 786-O xenograft model Biodistribution of CD70-TTC and an isotype control-TTC was analyzed 7 days after single i.v. administration of each compound at a dose of 500 kBq/kg (protein dose of 0.36 mg/kg). **(A)** Accumulation of ^227^Th expressed in % of injected dose per gram, decay corrected to the timepoint of injection in tumors and organs. **(B–C)** Accumulation of ^227^Th (Bq/g) and ^223^Ra (Bq/g), decay corrected to timepoint of euthanization in tumors and organs.

The amount of ^227^Th and ^223^Ra (the decay product of ^227^Th) in the tumor and in major organs seven days after treatment are presented in Figure 4. In animals treated with CD70-TTC, the tumors had decay corrected average activity of (9.66 ± 3.86) kBq/g ^227^Th corresponding to (122 ± 42) % of injected dose/gram (% ID/g) in the tumor. In comparison ^227^Th-uptake in organs was less pronounced, ranging from (19 ± 20) Bq/g for large intestine to (219 ± 98) Bq/g for the liver, corresponding to 0.3 and 2 % ID/g respectively. The measured activities for ^223^Ra ranged from below detection limits for liver and muscle to (207 ± 121) Bq/g in femur, rising to (523 ± 242) Bq/g in the tumors. In contrast, animals treated with the isotype control-TTC had an average activity for ^227^Th of (339 ± 271) Bq/g in the tumors (3 % ID/g), (74 ± 14) Bq/g in large intestine and (447 ± 57) Bq/g in liver, corresponding to 0.8 and 4.7 % ID/g respectively. Measured activities in muscle were (39 ± 4) Bq/g (0.6 % ID/g) and (570 ± 66) Bq/g in blood (5 % ID/g). For ^223^Ra, treatment with the isotype control resulted in an average concentration in tumor of (46 ± 15) Bq/g, and tissue concentrations ranged from not detected (liver and kidneys) to (369 ± 131) Bq/g in femur.

### *In vivo* anti-tumor activity of the CD70-TTC

The *in vivo* anti-tumor activity of the CD70-TTC was assessed in the subcutaneous 786-O xenograft mouse model with lesion dimensions averaging 150 mm^3^. The CD70-TTC was administered as a single intravenous injection at activities of 50 kBq/kg, 100 kBq/kg, 300 kBq/kg and 500 kBq/kg at a fixed protein dose of 0.36 mg/kg antibody-chelator conjugate. Additional treatment groups included the isotype control-TTC dosed at 500 kBq/kg (0.36 mg/kg protein), vehicle-treated animals and non-radiolabeled CD70 antibody-chelator conjugate (0.36 mg/kg protein).

**Figure 5:**
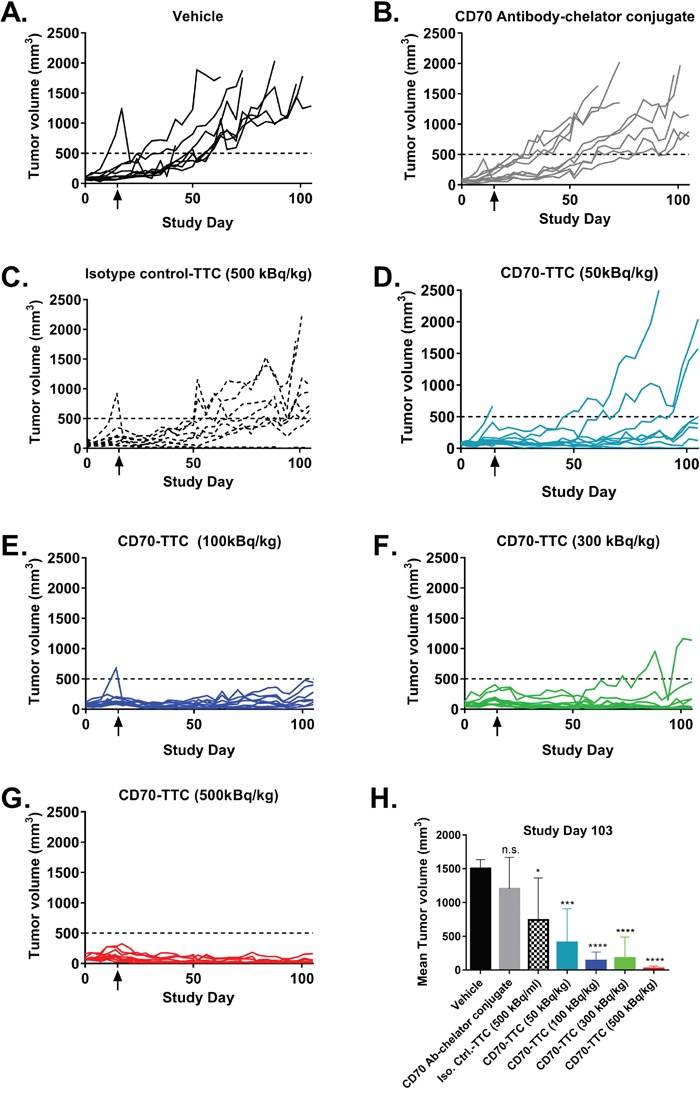
Individual tumor growth curves in the subcutaneous 786-O xenograft model At an average tumor size of 150 mm^3^, animals received a single i.v. injection (study day 15; indicated with an arrow). **(A)** Vehicle. **(B)** CD70 antibody-chelator conjugate. **(C)** Isotype control-TTC (500 kBq/kg at 0.36 mg/kg). **(D–G)** CD70-TTC (doses of 50, 100, 300 and 500 kBq/kg at 0.36 mg/kg). **(H)** Comparison of average tumor volumes at study day 103. Statistical analysis was performed using one-way ANOVA (Dunnett's testing) in comparison to vehicle (n.s., not significant; *, p < 0.05; **, p < 0.01; ***, p < 0.001; ****, p < 0.0001).

At study day 103 (three out of ten animals still alive in the vehicle treated group; humane endpoint was selected to be tumor volumes ≥ 1500 mm^3^) statistical analysis of tumor growth inhibition was performed. All CD70-TTC dose groups demonstrated statistically significant tumor growth inhibition when compared to the vehicle control group (see Figure 5 and Table 1). Remarkably, based on average tumor volumes, at a dose of 100 kBq/kg tumor stasis was observed, whereas at 300 and 500 kBq/kg measurable tumor regression was achieved. In contrast, animals treated with isotype control-TTC (500 kBq/kg) showed no statistical significant decrease in tumor volume compared to vehicle demonstrating the specificity of the CD70-TTC. Analysis of the individual target lesions based on the RECIST criteria [32] (Table 1) showed that two animals treated with CD70-TTC at a dose of 50 kBq/kg had complete remissions (CR), while eight animals had progressive disease (PD). Further, at the dose of 100 kBq/kg, four animals had CR's whereas 6 had PD. At the dose level of 300 kBq/kg, five animals had PD, one animal had stable disease (SD) and four animals showed CR’s. At the top dose of 500 kBq/kg, one animal had a PD, one animal a SD, 1 animal a partial response (PR) and seven animals showed CR’s. In contrast, seven animals treated with isotype control-TTC (500 kBq/kg) had PD and 3 animals showed CR’s.

**Table 1:** Tumor volumes, median survival times (MST), evaluation of target lesions and changes in body weight (BW)

	Vehicle	CD70 Antibody-chelator conjugate	Isotype control-TTC	CD70-TTC			
			500 kBq/kg	50 kBq/kg	100 kBq/kg	300 kBq/kg	500 kBq/kg
**Average tumor volume [mm^3^]**	1502 ± 132	1204 ± 464	953 ± 532	405 ± 502	139 ± 128	176 ± 313	24 ± 33
**T/C**	n/a	0.8	0.69	0.27	0.09	0.12	0.02
**Statistical significance^a^**	n/a	n.s.	*	***	****	****	****
**MST (days; end of study)**	86	91	113	n/a	n/a	n/a	n/a
**Survival**	0/10	1/10	0/10	6/10	9/10	8/10	10/10
**Statistical significance^b^**	n/a	n.s.	*	**	***	****	****
**Target lesion evaluation**	10 PD	9 PD/1 CR	7 PD/ 3 CR	8 PD/ 2 CR	6 PD/ 4 CR	5 PD/1 SD/ 4 CR	1 PD/ 1 SD/ 1 PR/ 7 CR
**BW in % (end of study)**	111	118	107	115	116	115	106
**Survival**	0/10	1/10	1/10	6/10	9/10	8/10	10/10
**Statistical significance^a^**	n/a	n.s.	****	n.s.	n.s.	n.s.	****

Average tumor volumes and treatment over control ratio (T/C) were determined at study day 103. MST, evaluation of target lesions based on RECIST criteria and changes in BW were analyzed at the end of study.

n/a, not applicable; n.s., not significant; ^a^, One-way ANOVA (Dunnett's test;); ^b^, Log-Rank (Mantel-Cox) test; *, p < 0.05; **, p < 0.01; ***, p < 0.001; ****, p < 0.0001; PD, progressive disease; SD = stable disease; PR, partial response; CR, complete response.

**Figure 6:**
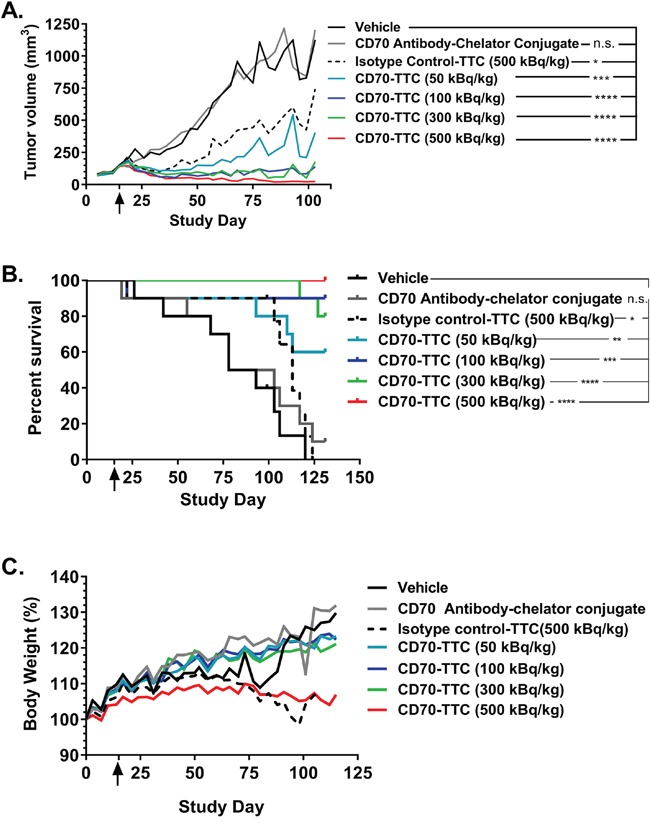
Average tumor growth inhibition, survival analysis and body weight changes Start of treatment is indicated with an arrow in each plot (study day 15). **(A)** Average tumor growth inhibition until study day 103. Statistical analysis was performed using one-way ANOVA (Dunnett's testing) in comparison to vehicle (n.s., not significant; *, p < 0.05; **, p < 0.01; ***, p < 0.001; ****, p < 0.0001. **(B)** Survival plot analysis of animals sacrificed due to humane endpoint (tumor volume > 1500 mm^3^). Animals were either treated with vehicle, CD70 antibody-chelator conjugate, isotype control-TTC (500 kBq/kg; 0.36 mg/kg) or with CD70-TTC at the indicated doses (50, 100, 300 or 500 kBq/kg; 0.36 mg/kg). The median survival time (MST) for the vehicle control group was determined to be 86 days and the MST of the CD70 antibody-chelator conjugate was 91 days, whereas the MST for animals treated with isotype control-TTC was 113 days. The MST for groups receiving CD70-TTC could not be determined. Data were analyzed using Log-Rank (Mantel-Cox) analysis (n.s., not significant; *, p < 0.05, **, p < 0.01; ***, p < 0.001; ****, p < 0.0001). **(C)** Changes in body weight, expressed in %.

The above described effect on tumor growth inhibition (Figure 6A) resulted in a clear dose dependent increase of median survival as illustrated in Figure 6B, and summarized in Table 1. At the end of study day 131 there were no survivors in either the vehicle or isotype control-TTC groups, with only one animal remaining in the group treated with the non-radiolabeled CD70 antibody-chelator conjugate. In contrast, 6, 9, 8 and 10 animals survived in groups treated with 50, 100, 300 or 500 kBq/kg of CD70-TTC respectively. Furthermore, based on the recorded body weight gains, there were no overt signs of toxicity (Figure 6B; Table 1).

**Figure 7:**
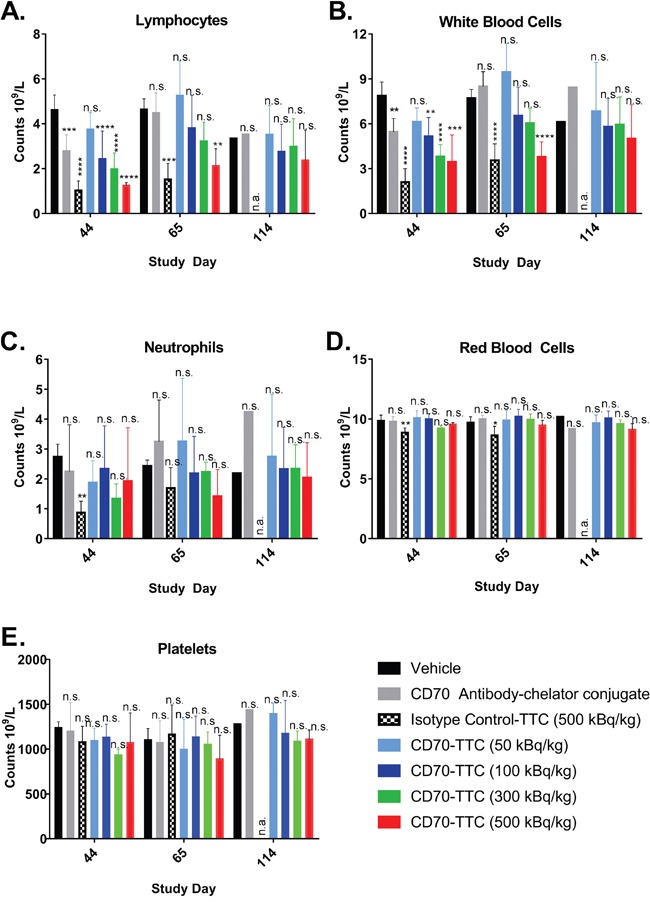
Hematology during course of *in vivo* efficacy study Blood chemistry analysis was performed at study day 44, 65 and 114. Statistical analysis was performed using one-way ANOVA (Dunnett's testing) using vehicle as baseline (n.s., not significant; *, p < 0.05, **, p < 0.01; ***, p < 0.001; ****, p < 0.0001). At day 114, no animals treated with isotype control-TTC were alive and therefore blood analysis was not applicable (n.a.). **(A)** White blood cell counts. **(B)** Lymphocytes. **(C)** Neutrophils. **(D)** Red blood cell. **(E)** Platelets.

To evaluate general treatment-related toxicity in addition to body weights, blood samples from individual animals from each group were analyzed during the course of the study. Results are presented in Figure 7. Dose-related suppression of neutrophils, lymphocytes and total white blood cells (WBC) were observed for animals treated with CD70-TTC at dosages of 300 and 500 kBq/kg, reaching statistical significance for lymphocytes and total WBC on study day 44 compared to the vehicle control group. For the isotype control-TTC, hematological depression on day 44 was even greater, reaching statistical significance for neutrophils, lymphocytes and WBCs. On day 65 there was partial recovery in all groups, and by day 114, complete recovery was seen in all surviving groups.

There were only minor reductions of red blood cells (RBCs), reaching statistical significance only in animals treated with isotype control-TTC (500 kBq/kg) at study day 44 and 65 compared to animals treated with vehicle (Figure 7D). No statistically significant differences in platelet counts were seen in any of the groups at any of the time points (Figure 7E).

## DISCUSSION

The current treatment options for primary RCC tumors are surgery (radical or partial nephrectomy), conventional chemotherapy, as well as stereotactic radiotherapy [33]. For metastatic RCC, therapies targeted at the vascular endothelial growth factor pathway have become the standard treatment. However, drug resistance to these agents has been reported, although the exact mechanisms are currently largely still unknown [34]. Therefore, new treatment modalities, such as the recently approved multi tyrosine kinase inhibitor cabozantinib [35] and immunotherapy-based treatments targeting the PD-1/PD-L1 axis [36, 37] are needed to further improve the clinical outcome in RCC.

Due to the constitutive expression of CD70 on solid tumors and B-cell lymphomas, several CD70-targeting molecules including antibodies and ADCs have recently entered preclinical and clinical testing in NHL and renal cell cancer [24, 30, 38]. In addition, the CD70-CD27 receptor-ligand pair has been suggested as a promising target for immunotherapy combinations by stimulating anti-tumor immune response, for example in NSCLC [39, 40]. In the present work, we describe the generation of a CD70-TTC that utilizes the benefits of the high linear energy transfer of alpha particle emitters delivered to the tumor by an antibody.

The preparation of the CD70-TTC drug product comprises two main steps. Firstly, the chelator moiety is conjugated to the antibody, the resulting conjugate following purification and re-formulation can be radiolabeled directly with ^227^Th. All steps are performed at ambient temperature and under mild conditions with the aim of preserving protein function and avoiding formation of impurities such as complex aggregates. Indeed for the non-radioactive conjugate, aggregate levels (dimers) were found to be below 3 % and both ELISA and FACS assays demonstrated high affinity binding of the antibody-chelator conjugate comparable to the parental CD70 antibody. Furthermore, on incubation with ^227^Th for 20-30 minutes, the radiochemical purity of the resulting CD70-TTC was ≥ 95% as measured by iTLC. In addition, in *in vitro* cytotoxicity assays using the human derived renal cell cancer cell line 786-O, we observed a specific reduction in cell viability over time as compared to the isotype control-TTC. The specificity and mode of action (MoA) are therefore anticipated to depend on both the targeting effect of the antibody to CD70 to the tumor cells and the emission of high energy alpha-particles inducing DNA double strand breaks (DSBs) resulting in G2 cell cycle arrest and cell death. We have previously reported data on this MoA in a recent study using a TTC targeting the CD33 receptor [8]. This has also been reported for other alpha-particle emitting nuclides such as ^213^Bi [41] and therefore we would postulate a similar mode of action for the CD70-TTC.

In the biodistribution study performed in tumor bearing animals using the human renal cancer cell line 786-O, high levels of tumor uptake were measured with (122 ± 42) % of the injected dose per gram of the CD70-TTC retained in the tumor compared to only ∼ 3 % ID per gram for the isotype control-TTC, a 40 fold difference at day 7. In contrast, the measured accumulation of ^227^Th in normal tissues and blood were at lower levels in the CD70-TTC treated animals compared to the isotype control-TTC animals, most likely due to the greater target-mediated accumulation of the CD70-TTC in the tumor leading to more rapid blood clearance of CD70-TTC and reduced uptake into normal tissue. The high accumulation of ^227^Th in the tumor is driven by (a) the binding properties of the non-mouse cross-reactive CD70 antibody-chelator conjugate combined with (b) the high levels of CD70 target expression in this model (c) low levels of necrosis (ranging from not detectable to 14 %) and (d) high vascularity of the tumors (MVD ∼ 324/mm^2^). ^223^Ra concentrations in all tissues were less than for 227^Th^ with the exception of femur where slow accumulation of ^223^Ra activity was observed reflecting the release from the chelator and redistribution of ^223^Ra, a bone seeking radionuclide [4]. To better understand the pharmacokinetic properties of the CD70-TTC, accumulation of ^227^Th and ^223^Ra in the different organs at additional timepoints (early and later than day 7) will be needed.

Statistically significant *in vivo* potency was achieved at all doses tested with complete tumor growth inhibition observed at doses as low as 50 kBq/kg. Although doses of 500 kBq/kg of the isotype control-TTC reached some statistical significance when examining the average tumor volume, this can be explained by the enhanced permeability and retention (EPR) effect, a common feature of antibodies [42, 43]. The efficacy in this model also translated to an increase in median survival times of the treatment group compared to control animals.

Myelosuppression was observed as a dose–related depression of circulating neutrophils, lymphocytes and total white blood cells over the course of 114 days as compared to the vehicle-treated animals. However, this toxicity appeared to be reversible with partial recovery of neutrophils, lymphocytes and white blood cells evident at 65 days and complete recovery by day 114 post dosing. The reversible hematological depression is in agreement with the previously reported literature for ^227^Th-based radioimmunoconjugates [8, 44]. The higher depression of neutrophils, lymphocytes and total WBCs seemed to be more pronounced for the isotype control-TTC and might be explained by the difference in pharmacokinetic profile with the higher levels of ^227^Th circulating in blood resulting in greater exposure of the systemic lymphoid tissues and bone marrow. No impact on red blood cells or platelets was observed. Although these toxicological findings in mice are encouraging, further toxicological experiments in non-rodent species will be needed to further evaluate myelosuppression as it is well accepted that rodents show greater resistance to radiation, including external beam radiation. As such the LD50 dose for rodents has been described to be in the range of 500 to 1000 rad (depending on the age of the animals) [45] in comparison to an LD50 of 500 rad in humans [46]. As expected from previous studies, no major findings were recorded in renal toxicity caused by accumulation of the daughter nuclides of ^227^Th [44] in contrast to redistribution of ^213^Bi formed from decay of ^225^Ac-based radioimmunoconjugates [47].

In summary, the *in vitro* and *in vivo* preclinical data provided herein for CD70 expressing tumor cells of renal cell carcinoma origin supports the further development of a CD70-TTC for this indication. As recently presented the optimized chelator chemistry allows both conjugation and radiolabeling steps to be performed under mild conditions and is applicable to a wide variety of targeting moieties [6, 8].

## MATERIALS AND METHODS

### Cells

The human renal cell cancer cell line 786-O was obtained from ATCC (Manassas, VA, USA) and was authenticated using PCR fingerprinting by the provider. The cell line was maintained in an incubator with an atmosphere containing 5 % CO_2_ at 37°C using RPMI-1640 medium supplemented with 10 % (w/v) fetal calf serum (FCS) and 1 % (v/v) penicillin and streptomycin.

### Synthesis of the chelator

The 3,2-HOPO chelator was synthesized in 4 steps from the starting materials N1,N1′-(2-(4-nitrobenzyl)propane-1,3-diyl)bis(N1-(2-aminoethyl)ethane-1,2-diamine) and 3-(benzyloxy)-1-(2-(benzyloxy)ethyl)-4-(2-thioxothiazolidine-3-carbonyl)pyridin-2(1H)-one [6].

### Preparation and characterization of CD70 antibody-chelator conjugate

Conjugation of the 3,2-HOPO chelator (494 μg) to a (non-cross-reactive to mouse) anti-human CD70 antibody (IgG1; 40 mg) was achieved through NHS-coupling of the 3,2-HOPO chelator to the lysine residues present in the antibody at a molar ratio of 3:1, respectively, and incubation for 1h at 21°C in PBS, pH 7.0. Monomeric fractions of the resulting CD70 antibody-chelator conjugate were separated from high-molecular weight (HMW) fractions using a HiLoad 16/600 Superdex 200 prep-grade SEC column (GE Healthcare) in formulation buffer. An isotype antibody-chelator conjugate was prepared in parallel.

The chelator to antibody ratio (CAR) was determined using a SEC-UV based method. Antibody-chelator conjugates were injected on a TSKgel SUPER SW 3000 column (Tosoh) using PBS/0.3M NaClO_4_ as the mobile phase, and the absorbance of the antibody-chelator conjugates at 280 nm and 335 nm (corresponding to the absorbance of the chelator) within the monomeric fraction was recorded. The CAR value was calculated using the following formula:

$$\text{CAR}=\frac{\left(\varepsilon mAb335-(R*\varepsilon mAb280)\right)}{\left((R*\varepsilon c280)-\varepsilon c335\right)},$$

with εmAb being the extinction coefficient of the antibody at 335 and 280 nm, εc being the extinction coefficient of the chelator at 335 and 280 nm and R being the chelator to mAb area ratio determined from the chromatograms. The CAR was determined to be 1.0.

### Radiolabeling of CD70-TTC

^227^Th was harvested from an ^227^Ac generator and the resulting purified isotope was stored in a solution of 0.05 M hydrochloric acid, containing metal-free water. Before radiolabeling, ^227^Th was further purified from the daughter ^223^Ra (including its short-lived progenies) using a strong anion exchange material as described in [48]. Two-hundred and fifty micrograms of antibody-chelator conjugate in formulation buffer were mixed with added ^227^Th activities in the range of 0.2 – 2.5 MBq, and incubated at room temperature for 20 – 30 minutes. Radiochemical purities (RCP) were determined by instant Thin-Layer Chromatography (iTLC) and were consistently in the range of ≥ 95%.

### Binding to CD70 antigen by ELISA and FACS analysis

To compare the binding properties of the CD70 antibody with the CD70 antibody-chelator conjugate, binding experiments to recombinant human CD70 in ELISA were performed. A 96 well plate (NUNC; Maxisorp) was coated with 3.5 μg/ml of anti-FLAG M2 antibody (Sigma Aldrich) diluted in PBS and incubated overnight at 4°C. The next day, the plate was washed 3 times with 250 μl/well wash buffer (PBS with 0.1 % (v/v) tween) using a plate washer. ELISA wells were blocked with 250 μl/well of 4 % PBS-milk for 2h. The plate was washed 3 times with 250 μl/well wash buffer using a plate washer afterwards and recombinant FLAG-TNC-CD70 was added at a concentration of 0.1 μg/ml in PBS-milk 0.1 % (w/v) to each well. The plate was incubated for 1h at room temperature and washed as outlined above. Serial dilutions (1:3) of CD70 antibody and CD70 antibody-chelator conjugate at starting concentrations of 4 μg/ml were added to the wells and incubated for an additional hour to allow binding to the target. Bound samples were detected by addition of 100 μl/well of anti-human-IgG-HRP antibody (Southern Biotech) diluted 1:15 000 in PBS with 0.4 % (w/v) milk. The plate was incubated for 30 minutes (min), washed, then 100 μl of ABTS (Thermo Scientific Pierce) was added to all wells. The plate was incubated for 5-10 minutes at room temperature and analyzed by measuring the absorbance at 450 nm in a plate reader (Perkin Elmer). The EC_50_ values were calculated using GraphPad Software.

Binding properties were tested on CD70-positive human 786-O cells. Cells were harvested from culture using trypsin, spun down at 300 g for 5 minutes at 4°C and washed with flow buffer (PBS with 0.5 % (w/v) fetal calf serum (FCS)). Cells were pelleted again by centrifugation (300 g), suspended in flow buffer and 50000 cells/well transferred to a V-shaped 96 well plate. After centrifugation (5 min at 300 g at 4°C), serial dilutions (1:3) of CD70 antibody and CD70 antibody-chelator conjugate were added to the cells at a starting concentration of 50 μg/ml. Samples were incubated for 1 h at 4°C. Unbound samples were washed off by excess flow buffer with an intermediate centrifugation step (5 min at 300 g at 4°C). Remaining bound samples were incubated with a 1:50 dilution of anti-mouse IgG-PE (Biolegend) antibody for 1 h at 4°C in the dark. The cells were washed twice with ice-chilled flow buffer with an intermediate centrifugation step and the mean fluorescence intensity (MFI) was recorded using a Quanta SC MPL machine (Beckman Coulter). The EC_50_ values were calculated based on the recorded median fluorescence intensities (MFI), using GraphPad software, also used for additional evaluations.

### *In vitro* cytotoxicity of CD70-TTC

The 786-O cells were harvested from culture flasks, counted and seeded at a density of 100.000 cells/ml into 24-well culture plates. Cells were incubated for 3h at 37°C with activities of 2 and 20 kBq/ml of CD70-TTC or an untargeted isotype control-TTC, both radiolabeled at a specific activity of 50 kBq/μg. A non-radiolabeled CD70 antibody-chelator conjugate sample was incubated on cells in parallel. Cells were washed with fresh medium after treatment and seeded into a new 24-well culture plate. At days 3, 5 and 7, cells were harvested and the viability was measured using the CellTiterGlo kit (Promega). The viability of treated cells was expressed as % of control cells that had been cultured in medium only.

### Animal models

Animal studies were conducted in collaboration with Pharmatest Services Ltd (Turku, Finland) with maintenance of rodents under barrier and pathogen-free conditions. Ethical approval was received from the National Committee for Animal Experiments (license number ESAVI-6057-04.10.03-2011). In all studies, animals received an intraperitoneal (i.p.) injection of an unrelated murine IgG2a antibody (200 μg/animal; UPC10; Sigma) 24 hours prior treatment to block unspecific spleen uptake [49]. Experiments were conducted using female NMRI nu/nu mice (Charles River, Germany) with a median weight of 18 to 25 gram and an average age of 7 to 10 weeks. Animals were injected with 5 × 10^6^ 786-O cells, suspended in 0.1 ml of 50 % matrigel (BD Biosciences). Subcutaneous tumor growth was monitored by measuring the tumor volume twice a week using a caliper and using the formula: V = 0.5 × (length + width)^2^. During efficacy studies animals were sacrificed by cervical dislocation upon reaching the humane endpoint with a tumor volume of 1.5 cm^3^ or a body weight loss ≥ 20%.

For biodistribution, tumors were grown until an average volume of 200-300 mm^3^ was reached. On the day of treatment, animals received a single intravenous injection of the CD70-TTC or an isotype control-TTC, both at a radioactive dose of 500 kBq/kg and a fixed protein dose of 0.36 mg/kg. Tumors and organs were harvested 7 days after dose administration and the radioactivity was counted using a high-purity germanium detector (HPGe) linked to an autosampler (Gamma Data). To identify ^227^Th and ^223^Ra, the GammaVision software and Npp32 analysis engine (Reg. Guide 4.16 detection limit method) were used. For ^227^Th measurement, the 235.96 keV (abundance 12.90%), 256.23 keV (abundance 7.00%), 329.85 keV (2.90% abundance), 286.09 keV (abundance 1.74%), 304.50 keV (abundance 1.15%), 334.37 keV (abundance 1.14%), and 299.98 keV (abundance 2.21%) gamma peaks were used. About 323.87 keV (abundance 3.99%), 338.28 keV (abundance 2.84%), 445.03 keV (abundance 1.29%), 269.46 keV (abundance 13.90%), 154.21 keV (abundance 5.70%), and 144.24 keV (abundance 3.27%) gamma peaks were used for ^223^Ra measurement. Thorium-227 counts were corrected to the time of injection and expressed as % of injected dose of ^227^Th. Further, ^227^Th and ^223^Ra counts were corrected to time of euthanization and expressed in Bq/g.

For efficacy studies, treatment was initiated at an average tumor volume of approximately 150 mm^3^. On the day of treatment, animals received a single intravenous injection of CD70-TTC, unlabeled CD70 antibody-chelator conjugate or vehicle (formulation buffer). CD70-TTC was dosed at radioactive doses of 50, 100, 300 and 500 kBq/kg at a fixed protein dose of 0.36 mg/kg. A separate group of animals was treated with an untargeted isotype control-TTC at a radioactive dose of 500 kBq/kg at a fixed protein dose of 0.36 mg/kg. Tumor growth was monitored by measuring the tumor volumes twice a week along with the body weights. Blood samples (200 μl) for hematology were taken from the saphenous vein into capillary tubes containing EDTA after heating the animals under a halogen lamp. The samples were mixed and the blood was analyzed (Siemens ADVIA 120).

Statistical analysis was performed using GraphPad software, applying one-way ANOVA (Dunnett's testing; tumor growth inhibition and body weights) and log-rank (Mantel-Cox) tests (survival analysis).

### Immunohistochemistry and autoradiography

Tumors from mice allocated to the biodistribution part of the study and treated with the CD70-TTC or the isotype control-TTC were harvested on day 7 and analyzed for target expression (CD70), microvessel density (CD34) and necrosis. Tumors were embedded in paraffin blocks, sectioned at 4 μm and stained with either anti-mouse CD34 (MEC 14.7, sc-18917, Santa Cruz Biotechnology Inc; 4 μg/μl) or with anti-human-CD70 antibody (clone #301731; R&D Systems; 25 μg/ml). For autoradiography, the sections were dewaxed then dipped in Ilford K5 emulsion (Polysciences Inc.), held in a light-proof box at room temperature for up to 3 days and processed according to the manufacturer's instructions. All sections were counterstained with Harris’ hematoxylin containing 0.6 % Eosin Y with 1 % Phloxine B and 2 % Orange G. For autoradiographic and histological assessment, stained slides were scanned with a Panoramic 250 Digital Slide Scanner (3DHistec Ltd, Hungary). All histological and autoradiographic procedures were conducted at the research facilities of Pharmatest Services Ltd (Turku, Finland).
